# Light Reduction Capabilities of Homemade and Commercial Incubator Covers in NICU

**DOI:** 10.1155/2013/502393

**Published:** 2013-10-29

**Authors:** Susan M. Ludington-Hoe, Amel Abouelfettoh

**Affiliations:** ^1^Frances Payne School of Nursing, Case Western Reserve University, Cleveland, OH 44120, USA; ^2^Faculty of Nursing, Cairo University, Cairo, Egypt; ^3^College of Nursing, King Saud bin Abdulaziz University for Health Sciences, Al Ahsa 31982, Saudi Arabia

## Abstract

Reduction of high-risk neonates' exposure to aversive light stimulation is an important component of developmentally supportive care. In neonatal intensive care unit (NICU), usually light is reduced by reducing the room's light level or by using incubator covers. Many types of incubator covers are in use, including homemade and commercial covers. A comparative study was used to determine the light reducing capabilities of 19 homemade incubator covers, 2 commercial covers, and 1 receiving blanket. The covers were tested by covering and uncovering an incubator and an oxygen hood in the NICU during daytime and nighttime lightings. The light reducing capabilities value was determined for each cover using an Extech light dosimeter when the cover was placed over and removed from an oxyhood, and an incubator. The study showed that the light reducing capability of the commercial covers was 91.2%, the homemade covers capability was 72.1%, and the receiving blankets capability was 55.1%. A significant difference between the commercial and homemade covers was found (*F* = 452.50, *P* < 0.00). Commercial incubator covers are the most effective covers to achieve light reduction; homemade covers can be effective if made large enough so that they completely cover all sides of the incubator.

## 1. Introduction

Reduction of the exposure of high-risk neonates to aversive light stimulation is an important component of developmentally supportive care [1–3]. Neonatal intensive care units (NICUs) usually reduce light by reducing the room's light level or by using incubator covers [4, 5]. Many types of incubator covers are in use, including homemade and commercial covers. Homemade covers are not standardized, and only one report of their light reducing capability during daytime and evening time could be found [6], and that report was based on a simulated level of light created in a windowless nursing school skills because reports of light reduction capability of homemade incubator covers in an actual NICU could not be found. 

The lighting in nurseries is known to be relatively constant [1, 5, 7], but light levels really are quite variable [8]. Two measures of light are referenced in the study presented here. A footcandle (ftc) is defined as a unit of illumination on a surface that is one foot from a point source of one candle; lux is defined as a unit of illumination that is equal to the direct illumination on a surface that is one meter from a uniform point source of one candle intensity or equal to one lumen per square meter (lumen/m^2^). The amount of light received by any infant varies from 15 to 2500 ftc (foot candles) or from 1.5 to 250 luxes, depending on his or her location in the nursery, the time of day, and the amount of sunlight present [9–11].

The light level that is appropriate for an NICU is still controversial [8] and may account for the persistently high levels of light in NICUs (Table 1). 9-10 luxes are thought to be needed to allow evaluation of infant skin color and perfusion [12]. The most recent Illuminating Engineering Society recommendations suggest ambient light levels of 1-2 luxes (10–20 ftc) with individual rheostat controls providing temporary increases in illumination for assessments and procedures [13]. Individual lighting creates a substantial change in ambient light, as does the use of halogen spot lights instead of fluorescent overhead lighting [12]. Light intensity is also reduced by 10% on average with double-walled incubators during normal nighttime electric hospital lighting [14]. 

Infants in the NICU spend weeks or months exposed to the NICU's light level. Research with animals has established the hazards of exposure to light at intensities similar to those found in the nursery [8, 15]. The effects of light on preterm infants have been reviewed extensively elsewhere [2, 16–18]. Exposure to fluorescent lighting greater than 60 ftc for preterm infants weighing less than 1000 gms has been associated with more retinopathy of prematurity (ROP) than exposure to 25 ftc or less [19]. The finding of increased retinopathy was not replicated in Ackerman and colleagues' study [20]. However, current thought is that high light levels do not affect ROP [21]. Altered visual functioning and arrested eye growth are important considerations for infants less than 750 grams, as these infants are experiencing a marked increase in ROP [22]. 

 Oxygen saturation remained stable with light reduction from 100 ftc to 5 ftc, and 22% of infants experienced a significant oxygen saturation decrease when light was increased from 5 ftc to 100 ftc [23]. Slevin and colleagues found that when NICU light was reduced during quite periods (switching off all the lights and pulling down all the blinds), infants' oxygen saturations increased by 0.25, and diastolic blood pressures and mean arterial pressures were significantly reduced by 2 mmHg [24]. Suppression of melatonin is associated with high illumination levels [25]. Sleep is also adversely affected by high levels of light [26]. In contrast, reduced light levels (using blindfolds for 10 hours per night) have been associated with increased alertness and improved respiratory stability [27] and a 75% reduction in infant movements and a 2 mmHg reduction in blood pressure [24]. Reducing visible light by wearing goggles has failed to improve weight gain, duration of oxygen therapy, duration of mechanical ventilation, or length of hospital stay in preterm infants [28], and covering incubators has failed to improve neurological and mental development or growth at 1 and 2 years after birth in a 32-week gestation preterm infants [29]. 

The incentive to reduce ambient light to foster sleep now exists. Preterm infants exhibited much more quiet sleep when ambient light levels were lowered by turning off several lights for 2-3 hours than when all lights in a room were left on [30]. When light reduction occurs regularly over part of the twenty-four hours of the day, the lighting condition is referred to as “cycled light.” Cycled lighting during lower lighting periods has been associated with decreased heart rate, decreased activity [31], decreased respiratory rate [32], improved sleep patterns, weight gain, and early establishment of rest-activity patterns that are in phase with the 24-hour light-dark cycle [26, 33]. Day-night cycling of light was found to increase sleep, decrease feeding time, and improve weight gain in 21 infants randomly assigned to a cycled light group when compared to 20 infants in a noncycled light group. Differences were still evident at 3 months after expected date of delivery [26]. 

Incubator covers have been identified as a means of cutting light but are disparaged for their “ill-fitting and unprofessional appearance” and for being made of fabrics that may prevent accurate evaluation of skin color [39]. Initially, infant flannel blankets were used in NICU to cover incubators. This practice expanded to the use of homemade covers of varying fabrics and size. Lee and colleges [6] evaluated the light reducing capacity of homemade quilted incubator covers by comparing the light level in the middle of an incubator in a nursing school skills laboratory when the covers were over the incubator and removed from the incubator. Quilted covers reduced the light by 37% when the incubator was covered. Light reductions in NICU were not measured nor were measurements taken during daytime and nighttime lighting conditions. Several commercial incubator covers are currently available. Some cover the entire incubator while others cover the top, sides, and back. Additionally, commercial covers come in different colors and are made of a variety of materials. Although these commercial covers are advertised as being able to “block out all excess light” and to “dim light,” no studies that examined the light reducing properties of commercial incubator covers or receiving blankets could be located. 

The current study was conducted to determine the light reducing capabilities of homemade crocheted, quilted, and flannel covers of a receiving blanket and of commercial incubator covers during daytime and evening time light levels in the NICU and to compare the light-reducing capabilities of homemade covers and a receiving blanket to commercial incubator covers during daytime and evening time light levels in a NICU.

## 2. Materials and Methods

A comparative, repeated measures study was conducted in a six-bed nursery (three to a side) in a tertiary care NICU in the first week of September in Northeast Ohio. The nursery has fluorescent lighting and one large external window with thin aluminum blinds; the window faces South onto a walkway between two tall buildings, and the NICU is on the second floor. For daytime lighting conditions, blinds were entirely open, 2 overhead fluorescent lights in the middle of the room were on, and six soffit lights over the wall-hugging counter (and directed away from the incubators) were on, providing an ambient lighting level of 628 luxes on the window side of the incubator used for testing. For nighttime conditions, the blinds were also open, overhead lights were turned off, and only two of six soffit lights over the counters were on, providing an overall ambient light level of 29 luxes on the window side of the one incubator used for testing. Reduced lighting conditions are commonly encountered during night, defined as 8:00 pm to 5:00 am for the purposes of this investigation. During the day of data collection, no quiet time was scheduled because quiet times are associated with large reductions in light (i.e., from 254 luxes to 3.0 luxes [24]). 

 Placement of the incubator in the nursery and the position of the oxyhood and researcher were standardized to prevent inconsistent shadowing and lighting. The unoccupied incubator was placed in the closest bed spot to the window. All light recordings were taken within the incubator, where the infant's head would be positioned on the side of the incubator towards the center of the nursery. 

### 2.1. Research Questions

The study reported here examined the light-reducing effects of commercial and homemade incubator covers and addressed the following research questions.

 Q1: What is the percent of light reduction with homemade covers, a receiving blanket, and two commercial covers that are placed over an Ohmeda Ohio Care Plus incubator during daytime and nighttime?
 Q2: What difference, if any, exists in percent of light reduction between homemade covers, a receiving blanket, and commercially available incubator covers?
 Q3: What is the percent of light reduction with homemade, receiving blanket, and commercial incubator covers that completely occlude an oxygen hood during daytime and nighttime?

### 2.2. Instruments

The Extech light dosimeter has a light sensor connected by a 3-foot cable to the digital display. The light sensor sat in the center of the oxygen hood and inside the incubator with the cable extending outside so readings could be taken without disturbing the placement of the covers to get the reading. The light dosimeter (a photometer) was autocalibrated each time; it was turned on, requiring a two-minute warm-up period (five minutes was given to prevent measurement drift) and was given a fifteen-second interval between different lighting conditions to insure accurate readings. A switch on the front of the dosimeter permitted the value to be digitally displayed as lux or foot candles; lux was chosen. The dosimeter has been approved for medical purposes and has reliability greater than 90% established by the manufacturer. The sensitivity of the dosimeter is ±0.10 lumen/ft^2^. One researcher read the light levels as another recorded them after repeating the value to the reader, a measure taken to insure recording accuracy. 

*Incubator Covers*. Ten homemade crocheted covers of varying sizes, nine three-layered homemade quilts, three homemade flannel covers, one commercial single-layer flannel receiving blanket (16% polyester, 84% cotton of 35 × 29 inches and white with 3 light blue stripes of 1 inch width interspersed between six 1/4 inch light pink and blue strips at one end of the blanket), and two commercial incubator covers (white with 1/4 inch diameter light purple, aqua, and pink hearts randomly spaced across the entire surface) (Children's Medical Ventures Incubator Cover Models, Boston, Mass, USA) were evaluated. The commercial incubator covers differed in that one (Model 6812315) did not cover the portholes and incorporated Velcro closures; the other (Model C100/450XL Incubator) had no open porthole areas (no flaps at all only a solid cover) and snaps for closure. Both commercial covers were quilted incubator covers.

### 2.3. Outcome Measures

#### 2.3.1. Light Reducing Capability

 Lighting was measured in lux as lumen/m^2^ [40]. The level of light is called illuminance and was measured by the light dosimeter. Light reducing capability was defined as the percent of light reduced by the covers (covered light level/uncovered light level) measured in lux.

#### 2.3.2. Procedure

The study was conducted from 11:30 am to 5 pm for daytime assessment and from 8:00 pm to 12 midnight for nighttime assessment. Data were collected in one day. 

 Homemade covers were collected from the large bin in the NICU. Detailed descriptions and measurements of each cover were recorded (Tables 2(a), 2(b), and 2(c)). The covers were taken to an incubator next to the window in one room of the NICU nursery. As most covers did not completely cover the incubator, determination of light-filtering abilities value over an oxyhood, which could be completely covered, was first performed. The oxygen hood was placed inside the unoccupied incubator described previously, at the place where an infant's head would be. The Extech light dosimeter (Model no. 401025; Extech Instruments, Waltham, MA, USA) was warmed up for five minutes with the light sensor in the center of the uncovered oxygen hood. Next, ten baseline measurements 15 seconds apart were taken from the center of the oxygen hood to determine mean ambient light. The hood was then completely enclosed by one of the covers, and after waiting 15 seconds for the meter to recalibrate, one light reading was taken. The hood was then uncovered, and the procedure with the same cover was repeated nine times more to obtain a mean light-filtering value based on ten readings for each cover. After data collection for the first cover was complete the procedure was repeated for each additional cover.

Next, the light reducing capability of the covers over an incubator was determined using the same procedure to acquire 10 baseline measurements of ambient light level inside the incubator (the meter was placed where the infant's head would be in the Ohmeda Incubator Plus double-walled incubator). A cover was then placed over the top edge at the “head side” of the incubator, and the light level was recorded. The incubator cover was then removed and immediately replaced and the light level was recorded. This procedure occurred ten times for each cover with 15-second intervals between each measurement. After determining light reduction of each homemade cover, two commercial incubator covers were put through the same procedure as was in one hospital receiving blanket. 

## 3. Results

Each of the covers was qualitatively described and measured (Tables 2(a), 2(b), and 2(c)). The area of each cover was also calculated. All covers varied in color hue, predominant color (dark colors did not predominate, the light colors did), length, width, density of the weave or material, and in their dimensions, reflecting possible variation when multiple people are engaged in producing quilts and when guidelines for the quilt's production are not given. The majority of the crocheted covers were too small to occlude any porthole and/or incubator side as they were the smallest of all the covers, and the crochet weave was loose in all of them. Most of the quilts were able to cover the nearest porthole the infant's head but did not go down the sides of the incubators nor simultaneously cover any other porthole. All of the homemade covers were smaller than the commercial covers and therefore did not cover the incubator as completely as the commercial covers. Thus, the area covered, in descending order, was 1902.27 (±57.94) inches by commercial incubator covers, 1474.80 (±243.30) inches by quilts, 1240.20 (±122.40) inches by flannels, 994.08 inches by the one commercial receiving blanket, and 382.70 (±159.20) inches by the crocheted covers. However, there was one crocheted cover that was larger than the others (no. 4), and when this outlier was removed, the mean area covered by crocheted blankets was reduced to 332.65 ± 14.79 inches. 

Ten recordings were obtained of ambient light level for daytime and nighttime lighting when the oxygen hood and incubator were covered and then uncovered, yielding ten values of percent of light reduction for each cover. Percent of light reduction for each cover over an incubator under day and nighttime conditions is conveyed in Table 3. Commercial incubator covers reduced light by 83%, followed closely by quilted (69%) and flannel (67%) covers. The receiving blanket reduced light by 47%, and crocheted covers reduced the least amount of light (44%). Light reduction capabilities over an incubator during night time were similar (Table 3). 

Light reducing capabilities over the oxygen hood are reported in Table 4 for daytime and night time conditions. During the daytime, commercial incubator covers reduced light by 99%, crocheted by 93%, flannels and quilts by 88%, and the receiving blanket by 62%. Light reducing capabilities over the oxygen hood changed with night time conditions: commercial incubator covers reduced light by 95%, flannels by 86%, quilts by 82%, crocheted by 72%, and commercial receiving blanket by 58%. 

Differences between the types of covers were analyzed using ANOVA and Tukey post hoc tests. The data from the one receiving blanket was removed from the ANOVA calculation. During daytime, percent of light reduction was significantly different between the covers when placed over an incubator (*F* = 16.99; *P* = 0.00), but it was not significantly different when placed over an oxygen hood (*F* = 2.43, *P* = 0.10). Commercial incubator covers reduced incubator daytime lighting significantly more than crocheted (*P* = 0.000); quilted covers reduced light more than crocheted (*P* = 0.000); flannels reduced light more than crocheted (*P* = 0.008); and crocheted covers reduced significantly less light than all other covers (*P* < 0.01). 

Under night time conditions, covers differed significantly in the ability to reduce lighting over an incubator (*F* = 16.69; *P* = 0.00) and over an oxygen hood (*F* = 3.26; *P* = 0.04). Crocheted covers reduced lighting less than quilted (*P* = 0.00), flannel (*P* = 0.01), and commercial incubator covers (*P* = 0.00) over an incubator. The difference in oxygen hood light reduction was between crocheted and commercial incubator covers, but the difference only approached statistical significance (*P* = 0.07). 

### 3.1. Open Weave versus Closed Weave

Two-tailed *t*-tests were conducted to determine light reducing difference between the translucent “open weave” crocheted covers (plus the receiving blanket which was quite thin and translucent too) and the flannel and quilted “closed weave” covers (Table 5). Closed weave covers reduced significantly more light than open weave covers in both daytime and night time conditions for the incubator and in night time conditions for the oxygen hood. 

## 4. Discussion

 The endpoints of the study were to determine the light reducing capabilities of homemade incubator covers and compare those capabilities to those of commercial incubator covers and a receiving blanket. Three types of homemade covers were assessed, crocheted, quilted, and flannel covers made by volunteers for a metropolitan, university-based neonatal intensive care unit. Crocheted covers reduced significantly less light than all other covers when they were palced over an incubator under day and night time conditions. The receiving blanket performed nearly as badly as the crocheted covers. Quilted and flannel covers performed relatively similarly to each other. Commercial incubator covers reduced the most light as compared to homemade covers and receiving blanket, but the difference in light reduction between commercial incubator covers and quilted and flannel covers failed to reach statistical significance. The data lead us to speculate that it is not the quality (weave or opacity) of the cover that reduced lighting, but the cover's size. Covers with an area equal to or greater than 1200 inches (30 × 40 inches) covered enough of the incubator to make them as effective in light reduction as commercial covers. The flannel and quilted covers were sufficiently large to meet this critical area. The receiving blanket's area was less than 1000 inches, and its light reducing capabilities were close to those of the poorest performing crocheted covers under daytime and night time conditions. 

 The data derived from testing the covers over an oxygen hood also support the proposition that the area that is covered determines light reducing capability better than the quality of the cover. All covers were able to occlude the oxygen hood completely, increasing the amount of light reduction of each cover. The light reducing capabilities of all covers when placed over an oxygen hood did not differ significantly, even though crocheted covers were loosely woven and had light penetrating holes in them. The data suggest that when the item can be completely covered by the homemade cover, open weave homemade covers are as effective as close weave homemade covers. 

 The incentive to cut light to improve developmental outcome of premature infants and to reduce ROP is a matter of question. A reduction in light and persistent nesting did not affect neurological and developmental outcomes at one and two years after birth in infants born at <32-week gestation [29]. In relation to ROP, a Cochrane review of randomized controlled trials of early light reduction (within seven days following birth) on the incidence of acute or poor outcomes of retinopathy of prematurity (ROP) in very low birth weight infants revealed no reduction in the incidence of ROP [41]. Yet, mechanisms by which light exposure can be detrimental exist. The pathophysiology of ROP in premature infants is understood to start with the injury to the incomplete developing of retinal capillaries. Damage could potentially occur before or during birth, but it is thought to primarily occur in the days following delivery when ambient light may reach the retina. Once the developing vessels have been damaged, it is hypothesized that the retina responds by producing vascular growth factors that stimulate new vascularization [41]. Oxygen free radicals are considered one cause of the injury of developing retinal capillaries in the premature infant [42]. Energy from light striking the retina may induce or increase the number of oxygen free radicals in the retina, particularly in the face of high levels of tissue oxygen. Numerous animal studies have demonstrated retinal injury from light, but these injuries have been due to other parts of the retina than the blood vessels (as is the case of infants) [42, 43]. Using incubator covers to decrease retinal ambient light exposure in premature infants to reduce the incidence of ROP is not supported by the existing evidence.

 Further research is needed to examine the influence of color and hue intensity on light reduction capabilities, as well as the effect of multiple washings. Confirmation by research is also needed to determine the effect of long-term use of incubator covers on infant development, especially regarding to visual development which matures rapidly as the infant approaches term age. Whether covers should be used continuously or intermittently should also be investigated, as continuous reduction of light and other sensory input may make it more difficult for the preterm infant to establish circadian rhythms [44]. Light reduction is still a major part of individualized developmental care for premature infants [5, 8, 45], and in concert with other elements of developmental care—that is, minimal interruptions, individualized caregiving based on infant organization, and skin-to-skin contact—may have a positive influence on the need for respiratory support, motor development, weight gain, and length of hospital stay [44]. Based on the findings reported here, commercial incubator covers are the most effective covers to achieve light reduction; homemade covers can be effective if made large enough so they completely cover all sides of the incubator. Closed weave covers will be more effective than open weave covers. 

## Figures and Tables

**Table 1:** Light levels in NICU and with different types of lighting.

Year	Light levels	Source of data
1960s	10 foot candles (100 luxes)	Glass et al., 1985 [19]

1980s	90 foot candles (900 luxes)	Glass et al., 1985 [19]

1981	Mean = 53 foot candles (530 luxes) Range = 34–140 foot candles (344–1400 luxes)	Gottfried et al., 1981 [34]

1990s	19–148 foot candles (192–1488 luxes)	Glotzbach et al., 1993 [35, 36], Robinson et al., 1990

1998	During day: mean = 29 foot candles (290 luxes) During evening: mean = 34 foot candles (340 luxes) During night: mean = 22 foot candles (220 luxes)	Gray et al., 1998 [37]

2001	Individual lighting decreases daytime ambient light level from 20 foot candles (200 luxes) to 1.5 foot candles (15 luxes) Fluorescent lights provide 19 foot candles (193 luxes) Halogen spot light on “maximum” provides 19-20 candles (193–202 luxes) Halogen spot light on default provides 1.2 foot candles (12 luxes)	Walsh-Sukys et al., 2001 [12]

2002	>646 luxes (60 footcandles)	American Academy of Pediatrics (2006) [38]

**Table:** (a)

Cover crochet	Length (inches)	Width (inches)	Total area (inches)	Weight (Kg)	Color side 1 (1), side 2 (2)
1	18.60	17.00	316.20	.100	Solid royal blue
2	19.10	17.20	328.52	.115	5 stripessolid navy blue royal blue, grey, and hunter green
3	18.30	17.40	318.42	.135	Solid light beige
4	35.60	23.40	833.04	.300	5 stripesrust, salmon, dark brown, cream, and dark green
5	20.00	18.00	360.00	.140	Solid sage and grey stripes
6	18.70	16.50	308.55	.105	Solid royal blue
7	18.60	18.40	342.24	.130	Solid red, rose, and brown stripes
8	20.20	16.40	331.28	.130	Solid heather and light beige stripes
9	20.70	17.50	362.25	.150	Solid sage
10	19.20	17.00	326.40	.100	Solid royal blue

Mean	20.90	17.88	382.70	.141	
SD	5.22	2.03	159.20	.059	

**Table:** (b)

Cover quilt	Length (inches)	Width (inches)	Total area (inches)	Weight (Kg)	Color side 1 (1), side 2 (2)
1	42.60	40.10	1708.30	.295	(1) Lime green, (2) lime green, mustard, white, and brown in floral pattern
2	42.00	34.00	1428	.500	(1), (2) Pink/red/hunter green/yellow patchwork with yellow and white flowers on white with fringed edge
3	34.00	33.00	1122	.900	(1) White, (2) snoopy figures on cream background
4	41.00	34.00	1394	.280	(1) Rust with small, white dots, (2) rose and green flowers
5	36.60	36.30	1328.60	.340	(1) Beige/cream check (2) Beige, beige/cream squares
6	36.50	36.20	1321.30	.295	(1) Pink with small white stars (2) alternating squares of pink, star fabric, and solid light pink
7	44.70	40.50	1810.40	.495	(1) Light blue/white stripes (2) Same as side 1 with strawberry shortcake circles
8	43.60	34.10	1486.80	.280	(1) White with pink/green/blue alphabet and angels (2) Squares of pink/green/blue letters
9	42.00	41.60	1747.20	.350	(1) White with pink/blue hearts/stars (2) Pink/yellow/blue clouds-white/lavender background

Mean	40.20	36.50	1474.80	.329	
SD	4.00	3.40	243.30	.068	

**Table:** (c)

Cover	Length (inches)	Width (inches)	Total area (inches)	Weight (Kg)	Color side 1 (1), side 2 (2)
Commercialclosed wave					
1-new	C = 37.70 R = 18.70 L = 19.10	C = 35.30 R =16.60 L = 16.70	C = 1330.81 R = 310.42 L = 318.97	.680	(1) Light grey (2) White with small purple, blue, purple, and pink hearts

2worn	C = 31.86 R = 16.37 L = 16.50	C = 37.13 R = 19.99 L = 20.25	C = 1182.96 R = 327.24 L = 334.13	.495	(1) Light grey (2) White with small purple, blue, purple, and pink hearts

Mean	MC = 34.78* MR = 17.54** ML = 17.8***	MC = 36.22 MR = 18.30 ML = 18.47	MC = 1256.89 MR = 318.83 ML = 326.55	.588	

Total mean Total SD				1902.27 ±57.94	

*MC: mean area of incubator that is covered. **MR: mean area of right end wall of incubator that is covered. ***ML: mean area of left end wall of incubator that is covered. C: center of incubator cover. R: right side cover of incubator cover. L: left side cover of incubator.

**Table:** (d)

Cover	Length (inches)	Width (inches)	Total area (inches)	Weight (Kg)	Color side 1 (1), side 2 (2)
Flannelclosed weave					
1	42.50	33.50	1423.80	.340	(1) Aqua, (2) yellow squares on green/white checked background
2	36.25	31.00	1123.75	.170	(1), (2) Small pink/white checks with yellow pony
3	4.37	34.13	1173.05	.360	(1) White, (2) random squares of pink, aqua, blue white, green, and bright yellow

Mean			1340.20		
SD			±122.40		

Receiving blanketopen weave					
1	34.88	28.5	994.08	.125	White with varying width pink and light blue stripes at one edge

**Table 3:** Percent of light reduced over incubator under daytime and nighttime conditions of all cover types.

Cover	Day				Night			
	*N*	Mean	SD	Range	*N*	Mean	SD	Range
Crochet	10	43.74	8.96	36–68	10	20.07	17.78	9–70
Quilt	9	69.28	11.45	57–86	9	65.34	16.94	45–90
Flannel	3	66.64	4.33	64–72	3	62.03	8.34	54–70
Receiving blanket	1	47.80			1	34.71		
Commercial covers	2	83.07	0.36	82-83	2	83.68	12.84	75–93

**Table 4:** Percent of light reduced over oxygen hood under daytime and nighttime conditions of all cover types and differences in light reduction between covers.

Cover	Day				Night			
	*N*	Mean	SD	Range	*N*	Mean	SD	Range
Crochet	10	92.60	2.05	89–95	10	71.56	13.38	53–94
Quilt^†^	9	87.20	9.31	75–99	9	82.04	10.84	68–95
Flannel^‡^	3	88.31	6.55	81–92	3	85.77	8.12	76–90
Receiving blanket*	1	61.56			1	57.69		
Commercial covers^±^	2	98.91	0.40	98-99	2	95.39	0.34	95.15–95.63

*Comparison not available because only one receiving blanket was tested, and no mean is available.

^†^Crochet versus quilt: daytime: *F* = 16.69, *P* = 0.000; nighttime: *F* = 5.32, *P* = 0.23.

^‡^Crochet versus flannel: daytime: *F* = 13.31, *P* = 0.008; nighttime: *F* = 7.62, *P* = 0.28.

^±^Crochet versus commercial: daytime: *F* = 15.71. *P* = 0.000; nighttime: *F* = 8.97, *P* = 0.07.

**Table 5:** Percent of light reduced over incubator and oxyhood by open weave and closed weave covers under daytime and nighttime conditions.

Source	*n*	Mean	SD	df	*t*	*P*
Incubator						
Daytime				20	−6.06	0.00*
Open	10	43.74	8.96			
Closed	12	68.62	10.01			
Nighttime				20	−6.37	0.00*
Open	10	20.07	17.78			
Closed	12	64.51	14.95			

Oxyhood						
Daytime				20	2.03	0.06
Open	10	92.6	2.05			
Closed	12	68.62	10.01			
Nighttime				20	−2.29	0.03*
Open	10	71.56	13.38			
Closed	12	82.97	10.01			

*P < .05.
